# Transmission and therapeutic modalities on condyloma acuminata in children: a case report

**DOI:** 10.11604/pamj.2025.50.101.45681

**Published:** 2025-04-11

**Authors:** Nissa Avina Pilar, Hasnikmah Mappamasing, Regitta Indira Agusni, Septiana Widyantari, Maylita Sari, Astindari Astindari, Afif Nurul Hidayati

**Affiliations:** 1 Department of Dermatology and Venereology, Faculty of Medicine, Universitas Airlangga, Dr. Soetomo General Academic Hospital, Surabaya, Indonesia

**Keywords:** Condyloma acuminata, children, trichloroacetic acid, sexual violence, case report

## Abstract

This case report presents a unique instance of condyloma acuminata in a one-year-old child, which adds to the scientific literature by highlighting the potential for non-sexual transmission in pediatric patients. The child exhibited a rapidly enlarging, skin-colored lump around the anus, initially a small bump that soon resembled a cauliflower. Acetowhite testing was positive, and histopathological examination revealed hyperkeratosis, acanthosis, and papillomatosis, leading to the diagnosis of condyloma acuminata. The main therapeutic intervention involved the application of 90% trichloroacetic acid (TCA), resulting in complete lesion resolution after two treatments, with no recurrence observed over a six-month follow-up period. This case emphasizes the urgent need for special attention to sexually transmitted infections (STIs) in children, considering the potential link to sexual violence. It highlights the critical, holistic role of venereology specialists, not only in providing effective and safe curative treatments but also in actively preventing further transmission and supporting vulnerable patients like children.

## Introduction

Condyloma acuminata (CA) is the most prevalent sexually transmitted infection (STI) worldwide, caused by the Human papillomavirus (HPV). Studies estimate that nearly 10-20% of the global population has been exposed to this virus. Human papillomavirus belongs to the papillomaviridae family, characterized as a double-stranded circular Deoxyribonucleic acid (DNA) virus, with some strains carrying oncogenic potential [1]. The most common HPV types causing CA are types 6 and 11 (accounting for 90% of cases), but the infection can pose a malignancy risk when caused by types 16, 18, 31, 33, 35, 39, 45, 51, 52, 55, 56, 58, and 59. Although HPV typically affects sexually active adults, cases in children have been documented, emphasizing the possibility of non-sexual transmission. Vertical transmission can occur during vaginal delivery from mothers with genital warts, and postnatal transmission can happen during diaper changes or bathing due to active warts on caregivers' hands or inadequate hand hygiene. However, thorough evaluation of all possible transmission routes, including sexual abuse, remains critical [1,2]. Multidisciplinary evaluation, encompassing thorough history-taking, physical, and psychological examination, is essential in pediatric STI cases, given the potential for sexual abuse. Treatment options for pediatric patients differ from those for adults due to children´s more sensitive skin, with additional considerations for co-existing conditions such as atopic dermatitis, which may contribute to recalcitrant CA, potentially leading to Buschke-Lowenstein tumors [1].

## Patient and observation

**Patient information:** a one-year-old female was brought to the Out-patient Dermatology and Venereology Department at Dr. Soetomo General Academic Hospital, Surabaya, with complaints of a skin-colored lump around the anus that had grown larger over the past two weeks. Initially presenting as a small bump, it rapidly increased in size, resembling cauliflower. The patient was delivered via uncomplicated vaginal delivery at full term, with the mother testing non-reactive for HIV during pregnancy and delivery. The patient is routinely cared for by elderly neighbors and their grandchild while both parents work. The mother is a laborer, and the father, an elementary school teacher, has been undergoing HIV treatment for a year.

**Figure 1:**
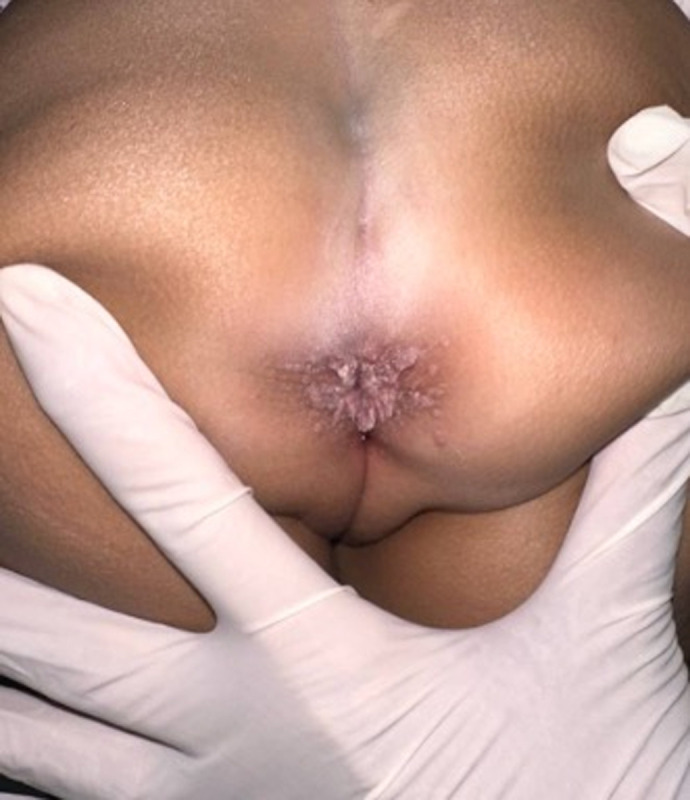
clinical findings during the initial visit

**Clinical findings:** on general physical examination, the patient was afebrile, with normal respiratory rate and heart rate, and appropriate weight and height for age. Dermatological examination revealed multiple skin-colored, pedunculated, verrucous papules resembling cauliflower around the anus (Figure 1). The surrounding skin was normal, with no signs of sexual abuse, such as lacerations or fissures. The acetowhite test was positive, showing whitish lesions. Histopathology confirmed hyperkeratosis, acanthosis, and papillomatosis in the epidermis. The patient was diagnosed with condyloma acuminata.

**Figure 2:**
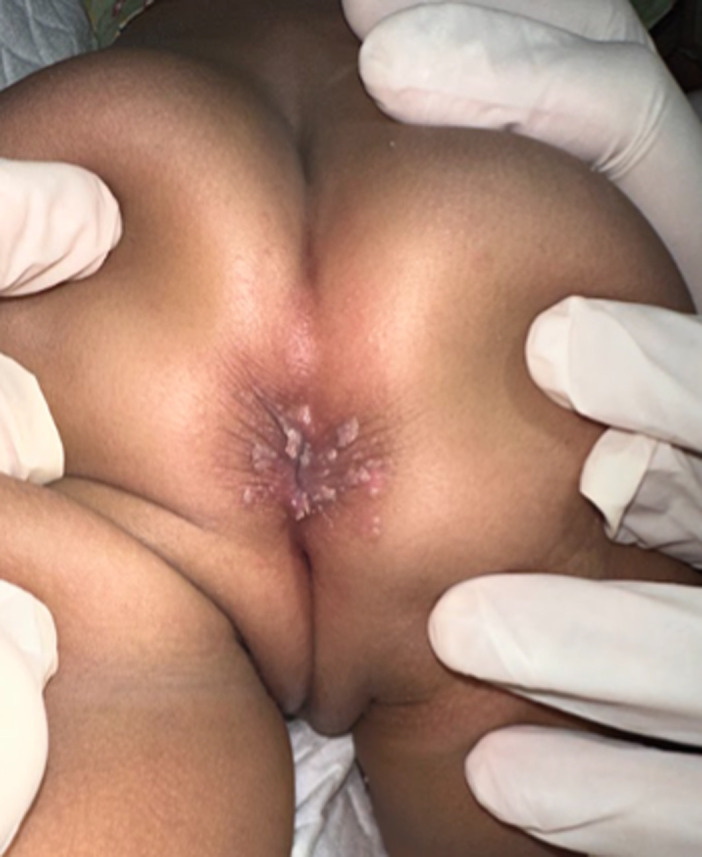
clinical presentation during the second visit

**Figure 3:**
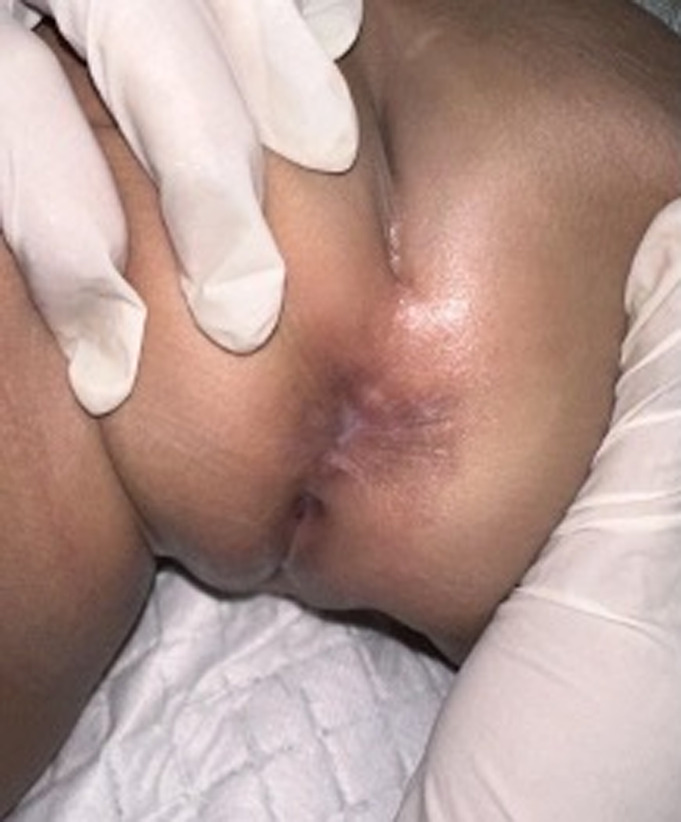
clinical presentation during the third visit after two applications of 90% trichloroacetic acid, one week apart

**Timeline of current episode:** the patient first presented with a rapidly growing, skin-colored lump around the anus, prompting the initial visit. On the first visit, physical examination, acetowhite testing, and histopathological analysis were conducted, leading to the diagnosis of condyloma acuminata. The initial treatment involved applying 90% trichloroacetic acid (TCA) directly to the warts, with vaseline album protecting the surrounding skin. At the second visit, one week later, partial resolution of the warts was noted without signs of infection, prompting a second application of 90% TCA (Figure 2). By the third visit, all lesions had resolved, leaving erythematous macules and hyperpigmentation but no ulcers or infections (Figure 3). The patient continued with regular follow-ups for six months, during which no recurrence of the lesions was observed.

**Diagnostic assessment:** the diagnostic approach included an acetowhite test, which was positive, indicating the presence of HPV lesions. Histopathology revealed characteristic features of condyloma acuminata, confirming the diagnosis. No challenges were faced in accessing diagnostic tests.

**Diagnosis:** the presumptive and final diagnosis was condyloma acuminata. No other diagnoses were considered.

**Therapeutic interventions:** the primary therapeutic intervention was topical 90% trichloroacetic acid (TCA) applied to the warts, with vaseline album protecting the surrounding skin. Additionally, 2% fusidic acid cream was prescribed to prevent infection, applied twice daily. Treatment was repeated after one week, resulting in complete resolution of the lesions.

**Follow-up and outcome of interventions:** during the six-month follow-up, the patient showed no signs of recurrence. The patient tolerated the treatment well, with no adverse effects or unexpected events reported. Follow-up assessments were conducted weekly for three weeks, then monthly for six months.

**Patient perspective:** while too young to provide direct feedback, the patient´s parents reported satisfaction with the treatment outcomes, noting rapid resolution of lesions and minimal side effects. They expressed relief that the child did not experience significant discomfort during the treatment.

**Informed consent:** it was obtained from the parents for all treatments and procedures, as well as for the use of disidentified patient information in this case report.

## Discussion

The present case demonstrates both strengths and limitations that are noteworthy. One of the strengths lies in its detailed account of a rare instance of pediatric condyloma acuminata (CA), specifically highlighting the potential for both vertical and horizontal transmission of the Human papillomavirus (HPV) in children. Most CA cases are associated with sexual transmission among sexually active adults, but the possibility of non-sexual transmission, particularly among children, is crucial to emphasize for pediatric care. Vertical transmission can occur during vaginal delivery from mothers with HPV, with studies suggesting three potential mechanisms: periconception (via HPV DNA in oocytes and sperm), prenatal (evidenced by HPV DNA in the amniotic fluid, umbilical cord, or placenta), and perinatal (direct contact with the infected vaginal mucosa). However, other studies have not consistently confirmed these findings, underscoring the need for further research [2-4]. Horizontal transmission, on the other hand, occurs through autoinoculation from another infected body part or heteroinoculation via contaminated caregivers´ hands during diaper changes or bathing, as well as through fomites, such as shared towels [5,6]. This case also raises awareness about the heightened risk of sexual violence in pediatric STI cases. In children, healthcare professionals must adopt a holistic approach, integrating curative treatment with preventive measures and protection strategies. The presence of symptoms such as erythema, lacerations, or fissures-especially at the 6 o'clock position on the perianal area-or signs of anal dilation can be suggestive of sexual abuse. Genital injuries in children can heal rapidly, making early and thorough evaluation crucial. Moreover, children may lack the ability to report incidents, emphasizing the importance of targeted education, careful history-taking, and diligent examination to ensure early detection and intervention [7,8]. The treatment approach for pediatric CA prioritizes effective and safe interventions with minimal side effects. Currently, there is no food and drug administration (FDA)-approved standard treatment for children under 12. Literature suggests that 80-90% trichloroacetic acid (TCA) is a viable option for pediatric CA, yielding satisfactory results with minimal side effects.1,3 In this case, two applications of 90% TCA over a one-week interval resulted in complete resolution of warts, with no associated erosion, ulceration, or infection. Importantly, no recurrence was observed over a six-month follow-up, further supporting the effectiveness and safety of this treatment modality in children.

## Conclusion

This case report highlights a rare occurrence of condyloma acuminata in a child, successfully treated with two sessions of 90% trichloroacetic acid (TCA), resulting in complete resolution with minimal side effects and no recurrence over six months. While non-sexual transmission is possible, assessing the risk of sexual abuse remains critical, especially in vulnerable children. A holistic approach involving thorough evaluation, education, and counseling ensures both curative and preventive care.
